# Sustainable Extraction Protocols for the Recovery of Bioactive Compounds from By-Products of Pomegranate Fruit Processing

**DOI:** 10.3390/foods13121793

**Published:** 2024-06-07

**Authors:** Gabriele Ballistreri, Margherita Amenta, Simona Fabroni, Nicolina Timpanaro, Giusy Maria Platania

**Affiliations:** Council for Agricultural Research and Economics (CREA), Research Center for Olive, Fruit and Citrus Crops, Corso Savoia 190, 95024 Acireale, Italy

**Keywords:** *Punica granatum*, by-products, wastes, sustainability, green extraction technologies, ultrasounds, microwaves, hydrodynamic cavitation, polyphenols

## Abstract

This study investigates sustainable extraction protocols for the recovery of bioactive compounds from by-products of various pomegranate (*Punica granatum* L.) cultivars, including Acco, Hicaz, Jolly Red, Parfianka, Valenciana, and Wonderful, generated during the industrial processing of the fruits. Advanced extraction technologies, including ultrasounds, microwaves, and hydrodynamic cavitation, have been compared to conventional extraction procedures and utilized to enhance extraction efficiency while also minimizing environmental impact. Water-based extraction methods have been utilized to promote the development of sustainable and eco-friendly processes. The comparison between conventional extractions and ultrasound-assisted extractions (UAEs) and microwave-assisted extractions (MAEs) demonstrated notable improvements in extraction yields, particularly for ellagitannins (punicalins, punicalagins, and ellagic acid) and total polyphenols, with increases ranging from about 45 to 200%. However, the increases directly comparing UAEs to MAEs ranged from about 4 to 6%. This indicates that while both UAEs and MAEs offer notable improvements over conventional extractions, the differences in extraction efficiency between the two advanced methods were relatively modest. These advancements were observed across various pomegranate cultivars, highlighting the versatility and effectiveness of these methods. Notably, hydrodynamic cavitation-based extractions (HC) emerged as particularly promising, consistently yielding the highest levels of bioactive compounds (ellagitannins and total polyphenols), especially when operated at higher frequencies. Compared to conventional extractions, HC exhibited substantial increases in extraction yields for Wonderful pomegranate by-products, surpassing the efficiency of both UAEs and MAEs (approximately 45 and 57% for UAE and MAE, respectively, versus about 80% for HC). Among these advanced techniques, HC has emerged as particularly promising, yielding the most favorable results and leading to significant improvements in the yield of bioactive compounds. When directly compared to UAEs and MAEs, HC increased extraction yields by over 20%. Furthermore, HC allowed for shorter extraction times. The Wonderful cultivar consistently exhibited the highest levels of ellagitannins and the highest total polyphenol content among all types of extraction procedures used, whether conventional or advanced. This highlights the great potential of the Wonderful cultivar in terms of bioactive compound extraction and underscores its significance in research and applications related to pomegranate processing and utilization. This study suggests that the implementation of these advanced technologies into extraction processes represents a significant advancement in the field, offering a promising avenue for the development of efficient and environmentally friendly extraction methods for obtaining valuable bioactive compounds from pomegranate processing by-products.

## 1. Introduction

The processing of pomegranate fruits involves various stages, from cultivation to industrial transformation, all aimed at harnessing its nutritional and commercial potential. Pomegranates (*Punica granatum* L.) are renowned for their rich phytochemical content, including polyphenols, specifically flavonoids, phenolic acids, and hydrolyzable tannins, mainly ellagitannins, which confer numerous health benefits [1,2]. The process begins with the selection of suitable cultivars and optimal growing conditions, followed by harvesting at the peak of ripeness to ensure quality. Subsequently, post-harvest handling techniques such as sorting, cleaning, and storage play pivotal roles in maintaining fruit integrity.

Processing methods for pomegranates encompass the extraction of juice as the main product, the production of concentrate, and the formulation of added-value products like jams, jellies, and dietary supplements. Each processing step influences the retention of bioactive compounds and sensory attributes of the final product. Furthermore, technological advancements have led to the development of innovative techniques such as cold pressing, enzymatic extraction, and microencapsulation, enhancing both efficiency and product quality.

The by-products of pomegranate fruit processing, such as peels, membranes, and seeds, constitute approximately 50% of the whole fruit [2,3]. There are numerous techniques for obtaining extracts from plant by-products. Traditionally, conventional methods rely on the use of chemical solvents. However, these methods pose challenges concerning waste disposal and environmental impact. Furthermore, some of the solvents used are not food-grade, necessitating purification steps during food production to mitigate residual toxicity. Green extraction techniques like ultrasound-assisted extraction (UAE) and microwave-assisted extraction (MAE) are increasingly being utilized to convert organic residues into recycled products with high added value and also offer numerous advantages over conventional solid–liquid extraction methods. These benefits include decreased extraction times and energy consumption, the elimination of chemical solvent residues, improved extraction efficiency, enhanced safety, and minimized environmental impact compared to traditional extraction methods [3,4,5,6,7].

Various sustainable extraction protocols have been explored for recovering bioactive compounds from by-products generated during pomegranate fruit processing. Among these methods, MAE emerged as being particularly effective, as it demonstrated high efficiency in recovering polyphenolic compounds [4]. Additionally, green methodologies utilizing pressurized liquids and deep eutectic solvents have been developed, enhancing the extraction of proteins and bioactive compounds from pomegranate peels [5]. Ultrasound-, microwave-, and enzymatic-assisted extraction techniques have also shown promise for bioactive compound recovery, especially in food matrices [6]. Furthermore, the utilization of emerging technologies and green solvents has been emphasized for extracting bioactive compounds from pomegranate peels, particularly for applications in the pomegranate juice industry [7].

Hydrodynamic cavitation (HC) is a promising alternative to UAE and MAE in terms of energy efficiency and scale-up capability (being a continuous process) for various applications, including extraction, emulsification, and wastewater treatment [8]. HC involves the generation of cavitation bubbles in a liquid medium through the rapid reduction in pressure, typically by passing the liquid through a constriction or a nozzle. As these cavitation bubbles collapse, they release energy in the form of shock waves, intense turbulence, and high temperatures. This phenomenon can disrupt cell structures and facilitate the extraction of bioactive compounds from organic materials, similar to UAE. One of the key advantages of HC over UAE and MAE is its potential for higher energy efficiency, as it typically requires lower power input for cavitation bubble generation. Additionally, HC systems can often be scaled up more easily than UAE and MAE setups, making them suitable for industrial-scale applications.

HC has been recently employed for the production of extracts from both pomegranate fruits and by-products [9,10]. However, in these studies, HC was not directly compared in terms of extraction efficiency with other extraction techniques.

For these reasons, this study investigates the employment of different advanced water-based extraction technologies, such as UAE, MAE, and HC, to enhance extraction efficiency in terms of yields of bioactive compounds (ellagitannins and total polyphenols). Moreover, the by-products resulting from the industrial processing of six different commercial pomegranate (*Punica granatum* L.) cultivars, including Acco, Hicaz, Jolly Red, Parfianka, Valenciana, and Wonderful, were subjected to water-based lab-scale extractions, UAEs, MAEs, and HC to assess their levels of bioactive compounds with the scope to identify the most promising cultivar for obtaining extracts with a high content of bioactive compounds.

## 2. Materials and Methods

### 2.1. Chemicals and Reagents

Folin–Ciocalteu reagent (FCR), sodium carbonate (Na_2_CO_3_), gallic acid, punicalin, a mixture of anomers (A + B mixture), punicalagin (A + B mixture), and ellagic acid were purchased from Sigma-Aldrich (St. Louis, MO, USA). All other chemicals were of analytical grade, and the solvents used for chromatography were of HPLC grade (Merck KGaA, Darmstadt, Germany).

### 2.2. Plant Material

The fresh pomegranate by-products (FPBs) deriving from the industrial transformation of six commercial pomegranate (*Punica granatum* L.) cultivars, including Acco, Hicaz, Jolly Red, Parfianka, Valenciana, and Wonderful, were provided by Oranfresh S.R.L. (Catania, Sicily, Italy). The FPBs, consisting of exhausted peels, membranes, and seeds, were dried in a ventilated oven at 40–50 °C until their moisture content was reduced to below 5% to have a stable material until the extraction procedures and subsequently cut into small pieces with a linear size of approximately 5–10 mm through a grinder (model Mx Type A505, Moulinex, Ecully Cedex, France). In all extraction procedures, the dried pomegranate by-products (DPBs) were utilized with a ratio of DPB to solvent of 1:10 (*w*/*v*).

### 2.3. Lab-Scale Extraction

Conventional lab-scale extractions were carried out using 0.2 kg of DPB suspended in 2.0 L of water with continuous stirring at 200 rpm for 80 min (min), maintaining the temperature under control and ensuring it remained below 40 °C. To ensure that the temperature during the extraction process was consistently maintained below 40 °C, a water bath with a precise temperature control system was employed. Specifically, a water bath (model TRM 750, ASAL S.r.l., Cernusco sul Naviglio, Italy) was used, allowing us to set and monitor the temperature accurately throughout the extraction process. The temperature was regularly checked using a calibrated digital thermometer to ensure it remained within the desired range. After the extractions, the aqueous extracts underwent filtration to remove any particulate matter, and the resulting filtrates were then utilized for subsequent chemical analyses.

### 2.4. Ultrasound-Assisted Extraction

The ultrasound system used for UAE was an ultrasound bath system (model C.E.E.M.30A, Sarl R.E.U.S., Drap, France), with an operating frequency of 24 kHz, a fixed maximum power of 1.25 KW, and a mechanical stirrer operating at 200 rpm. The UAEs were carried out using 2.0 kg of DPB suspended in 20.0 L of water under steady stirring for 8 extraction cycles of 10 min each, maintaining the temperature under control and ensuring it remained below 40 °C. To ensure that the temperature during the extraction process was consistently maintained below 40 °C, a calibrated digital thermometer was used to monitor the temperature within the desired range. Sonication was applied in cycles to prevent overheating and ensure efficient extraction. This cyclic application helped us maintain the temperature below the critical threshold. These methods were chosen to guarantee the preservation of the integrity of the extracted compounds, as excessive temperatures could potentially degrade sensitive components. After the extractions, the aqueous extracts underwent filtration to remove any particulate matter, and the resulting filtrates were then utilized for subsequent chemical analyses.

### 2.5. Microwave-Assisted Extraction

The experimental apparatus for MAE was a microwave-assisted extraction system equipped with a built-in water bath for temperature control (model 30 L MW, Sarl R.E.U.S., Drap, France) with an operating frequency of 2.45 MHz, a maximum power of 1.25 KW, a mechanical stirrer operating at 200 rpm, and a temperature feedback control water chiller (model Smart H150, LabTech Srl, Sorisole, Italy). Preliminary experiments were conducted at MW powers of 0.625 KW. However, these settings resulted in modest extraction yields and efficiencies. Therefore, all experiments were performed at the maximum power setting of 1.25 KW, which provided significantly better results (see Section 3.2). The MAEs were carried out using 2.0 kg of DPB suspended in 20.0 L of water under steady stirring for 8 extraction cycles of 10 min each, maintaining the temperature under control and ensuring it remained below 40 °C. The water bath component ensures that the temperature does not exceed the set limit of 40 °C, thereby preventing the thermal degradation of sensitive compounds. After the extractions, the aqueous extracts underwent filtration to remove any particulate matter, and the resulting filtrates were then utilized for subsequent chemical analyses.

### 2.6. Hydrodynamic Cavitation

The HC device used was a centrifugal cavitation system (model Tiny (T) lab unit Soldo, Three-ES S.r.l., Lazzate, Italy) with a maximum power of 5.5 KW and a volumetric pump with a maximum flow rate of 0.55 m^3^/h (type SFA90CA4, Liverani s.r.l., Lugo, Italy). The flow rate was set to 0.055 m^3^/h (0.917 L/min), which was determined to be the most effective for the experimental setup. This flow rate was chosen to ensure thorough extraction while avoiding any potential overheating that could occur at higher flow rates. Additionally, to ensure that the temperature during the extraction process was consistently maintained below 40 °C, a calibrated digital thermometer was used to monitor the temperature and ensure it remained within the desired range. The HC-based extractions were carried out using 2.5 kg of DPB suspended in 25.0 L of water for 8 extraction cycles of 10 min each performed at three different frequencies (40, 50, and 60 Hz), maintaining a controlled temperature below 40 °C. After the extractions, the aqueous extracts underwent filtration to remove any particulate matter, and the resulting filtrates were then utilized for subsequent chemical analyses.

### 2.7. Determination of Total Polyphenol Content

The Folin–Ciocalteu spectrophotometric method, usually used to determine total phenolics, was applied in this work also as an indicator of the antioxidant activity of pomegranate extracts, following procedures outlined in previous research [11]. Briefly, 0.1 mL of the filtered extract was diluted in 10 mL of distilled water; then, 1 mL of this solution was mixed with 5 mL of 10% Folin–Ciocalteu reagent and 4 mL of a Na_2_CO_3_ solution (7.5% *w*/*v*). The mixture was stirred for 2 h at room temperature in the dark. The absorbance of the resulting blue solution was measured spectrophotometrically at 765 nm. Analyses were performed in triplicate, and the concentration of total polyphenols was expressed as g of gallic acid equivalents (GAEs)/100 g of extract.

### 2.8. HPLC-PDA Analysis of Phenolic Compounds

Phenolic compounds were separated and quantified using HPLC-PDA analysis, following established protocols from previous studies [11,12]. Phenolic compounds were identified using retention time (RT) and UV–Vis spectra compared with those of pure standards. The quantification of each phenolic compound was performed at 378 nm, employing the corresponding standard as an external standard. Analyses were conducted in triplicate, and the results were expressed as g of compound/100 g of extract.

### 2.9. Statistical Analysis

The obtained data underwent statistical analysis using Statgraphic Plus version 4.1 (Manugistics Inc., Rockville, MD, USA). The one-way analysis of variance (ANOVA) was applied. Significant differences (*p* < 0.05) in the total polyphenol content and relative composition of phenolic compounds were assessed using Duncan’s multiple-range tests. All results were presented as the mean ± standard deviation (SD).

## 3. Results and Discussion

### 3.1. Lab-Scale Extractions

This study evaluated the levels of phenolic compounds (ellagitannins) and total polyphenols in the by-products resulting from the industrial processing of six different commercial cultivars of pomegranates (*Punica granatum* L.), namely Acco, Hicaz, Jolly Red, Parfianka, Valenciana, and Wonderful. Previous studies have examined the level of phenolic compounds in the by-products of different pomegranate cultivars, including Acco and Wonderful [13,14]. However, to the best of our knowledge, there have been no investigations regarding the levels of phenolic compounds in the Hicaz, Jolly Red, Parfianka, and Valenciana cultivars.

Additionally, there are few studies that have compared the amount of bioactive compounds in the by-products of different pomegranate cultivars using the same drying and extraction methodology [6,14]. Therefore, the findings presented in this study are particularly valuable due to their novelty in the field.

Conventional lab-scale extractions were conducted in order to assess the levels of bioactive compounds in the pomegranate by-products deriving from different pomegranate cultivars. These traditional solvent extractions were compared with advanced extraction technologies, including ultrasound, microwave, and hydrodynamic cavitation. All extraction methods operated under the same conditions of dried pomegranate by-products (DPBs)/solvent ratio (1:10, *w*/*v*) and extraction time (80 min). Water, being an environmentally friendly solvent for extraction, was employed in all extraction procedures.

**Figure 1:**
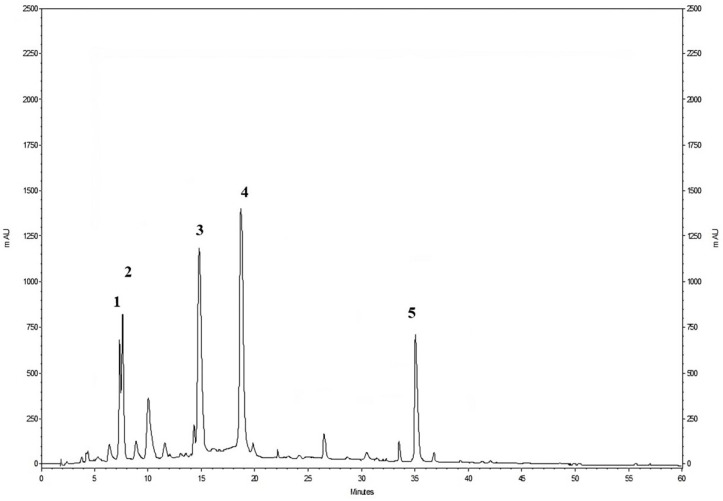
The HPLC profile of the phenolic compounds of a pomegranate by-product extract was detected at 378 nm. (1) Punicalin A; (2) punicalin B; (3) punicalagin A; (4) punicalagin B; (5) ellagic acid.

HPLC-PDA was used to evaluate and quantify the main phenolic compounds present in the extracts of pomegranate by-products (Figure 1), while the Folin–Ciocalteu methodology was employed to determine the total polyphenol content and also as an indicator of antioxidant activity. According to the literature, the main phenolic compounds found in pomegranate by-products are punicalagins, followed by punicalins and ellagic acid [6,15].

The levels of phenolic compounds (ellagitannins) and total polyphenols in the pomegranate by-product extracts obtained by lab-scale extractions are reported in Table 1. The highest levels of ellagitannins and the highest total polyphenol content were detected in the by-product extract of the Wonderful cultivar, followed by Jolly Red, Hicaz, Valenciana, Acco, and Parfianka. Among the cultivars analyzed, Wonderful exhibited the highest concentrations of punicalins (0.036 ± 0.001 g/100 g extract), punicalagins (0.126 ± 0.014 g/100 g extract), ellagic acid (0.029 ± 0.002 g/100 g extract), and total polyphenols (0.191 ± 0.011 g GAEs/100 g extract). The high levels of these bioactive compounds correlate with enhanced antioxidant activity, indicating the potential of Wonderful for applications requiring strong antioxidant properties. This suggests that Wonderful has the greatest potential for bioactive compound extraction, confirming findings by various authors who have highlighted its superior phytochemical content [16]. Comparatively, Parfianka showed the lowest values across all measured compounds, indicating a lower extraction efficiency. This could be attributed to the intrinsic properties of the Parfianka cultivar, which may possess a more robust cellular structure, thereby reducing the effectiveness of the extraction processes applied. Although pomegranate by-products are a substantial source of bioactive compounds, their quantity varies depending on the cultivar selected and the processing technology used. The values reported in Table 1 appear to be consistent with those found in the literature for various pomegranate by-products [6,14,15]. Any differences in values can be attributed to factors such as the growing conditions and extraction methodologies used.

### 3.2. Ultrasound- and Microwave-Assisted Extractions

**Figure 2:**
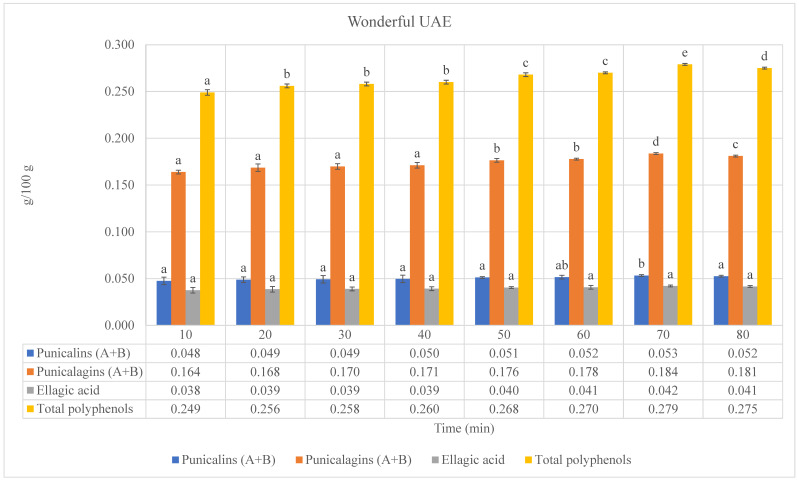
Phenolic compounds (punicalins, punicalagins, and ellagic acid), “a”, and total polyphenols, “b”, of Wonderful pomegranate by-product extracts obtained by ultrasound-assisted extractions (UAEs) “a”: values expressed as grams of standard compounds per 100 g of extract (g/100 g extract). “b”: values expressed as grams of gallic acid equivalents (GAEs) per 100 g of extract (g GAEs/100 g extract). Values are expressed as the mean ± standard deviation (SD). Mean values with different letters (a–d on the bars denote statistical differences (*p* < 0.05) among different extraction times for both phenolic compounds and total polyphenols.

**Figure 3:**
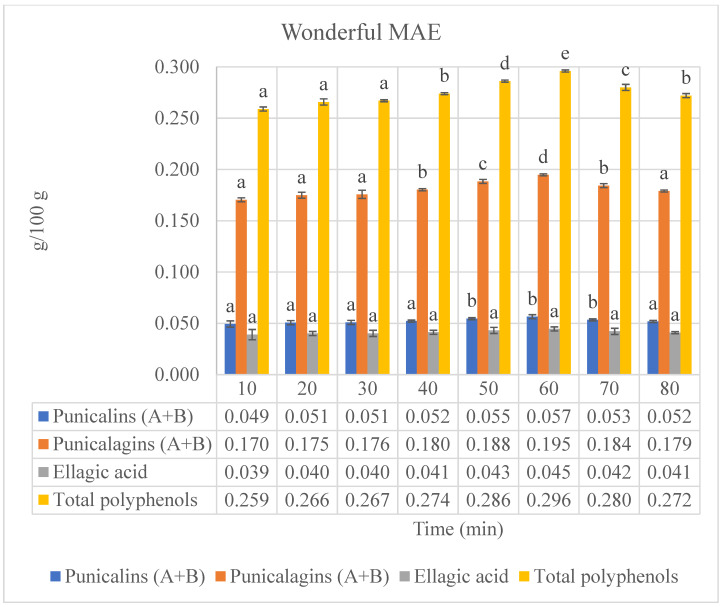
Phenolic compounds (punicalins, punicalagins, and ellagic acid), “a”, and total polyphenols, “b”, of Wonderful pomegranate by-product extracts obtained by microwave-assisted extractions (MAEs). “a”: values expressed as grams of standard compounds per 100 g of extract (g/100 g extract). “b”: values expressed as grams of gallic acid equivalents (GAEs) per 100 g of extract (g GAEs/100 g extract). Values are expressed as the mean ± standard deviation (SD). Mean values with different letters (a–e) on the bars denote statistical differences (*p* < 0.05) among different extraction times for both phenolic compounds and total polyphenols.

In order to compare a conventional solid–liquid extraction method to advanced extraction technologies, the pomegranate by-products of the different cultivars have been subjected to ultrasound- and microwave-assisted extractions. In both methods, the extracts were gathered at specific time intervals of 10 min each for 8 extraction cycles (80 min total) (the results are presented in Figure 2 and Figure 3 and in the Supplementary Materials (Figures S1 and S2)). The same trend regarding phenolic compounds (ellagitannins) and total polyphenols has been confirmed by both advanced extraction methods compared to lab-scale extractions; in fact, the highest levels of ellagitannins and the highest total polyphenol content were detected in the by-product extract of the Wonderful cultivar, followed by Jolly Red, Hicaz, Valenciana, Acco, and Parfianka. This order highlights the inherent differences in bioactive compound content among the cultivars, regardless of the extraction method used. The higher concentrations of these compounds in the Wonderful cultivar, including punicalins, punicalagins, and ellagic acid, which are known for their potent antioxidant properties, are directly associated with higher antioxidant activity, as confirmed by the Folin–Ciocalteu assay. Specifically, the high content of total polyphenols in Wonderful directly correlates with its superior free radical scavenging ability. This suggests that the genetic makeup of Wonderful facilitates the production and extraction of these phenolic compounds, contributing to its superior antioxidant properties [16]. The enhanced antioxidant activity of Wonderful underscores its potential for use in nutraceutical and functional food applications where antioxidant properties are desired.

The results confirmed that both advanced extraction methods (UAE and MAE) were more effective than conventional lab-scale extractions in recovering these antioxidant compounds. Several studies have reported the effectiveness of UAE and MAE in extracting phenolic compounds from various plant materials. UAE significantly enhances the extraction of polyphenols compared to conventional extraction methods [17]. This is attributed to the cavitation effects of UAE, which lead to improved cell wall disruption and solvent penetration, consistent with our results showing higher yields of bioactive compounds. Similarly, MAE is recognized for its efficiency in extracting phenolic compounds due to the localized superheating that enhances the solubilization of target compounds [18].

Our experimental approach ensured uniform conditions across all extraction techniques, utilizing the maximum power settings (1.25 KW) for both UAE and MAE. This consistency allows for a direct and fair comparison of the techniques. Both UAE and MAE demonstrated significant extraction efficiency, but there were notable differences. MAE consistently yielded higher concentrations of bioactive compounds compared to UAE. This can be attributed to the rapid heating and efficient energy transfer of microwaves, which enhance cell wall disruption and solvent penetration [18]. However, UAE also showed considerable effectiveness due to acoustic cavitation, which promotes cell rupture and enhances mass transfer [19]. The superior performance of MAE under these conditions aligns with literature findings that emphasize the efficiency of microwave energy in breaking down cell walls and facilitating the release of bioactive compounds, leading to higher extraction efficiency [20,21]. UAE, while effective, showed slightly lower efficiency due to its dependence on acoustic cavitation, which may not have been as uniformly effective across the plant material.

Comparing the maximum values of phenolic compounds (ellagitannins) and total polyphenols obtained by UAEs to those obtained by conventional extractions, it was found that the UAEs exhibited an increase in extraction yields. Specifically, punicalins, punicalagins, ellagic acid, and total polyphenols showed increases of 143.1%, 141.3%, 143.5%, and 139.6%, respectively, in the by-product extract of the cultivar after six cycles of extraction (60 min); punicalins, punicalagins, ellagic acid, and total polyphenols showed increases of 83.8%, 84.0%, 86.8%, and 83.1%, respectively, in the by-product extract of the cultivar Hicaz after six cycles of extraction (60 min); punicalins, punicalagins, ellagic acid, and total polyphenols showed increases of 68.7%, 70.2%, 72.3%, and 70.2%, respectively, in the by-product extract of the cultivar Jolly Red after seven cycles of extraction (70 min); punicalins, punicalagins, ellagic acid, and total polyphenols showed increases of 181.3%, 169.4%, 178.0%, and 172.9%, respectively, in the by-product extract of the cultivar Parfianka after six cycles of extraction (60 min); punicalins, punicalagins, ellagic acid, and total polyphenols showed increases of 124.5%, 122.7%, 119.2%, and 122.5%, respectively, in the by-product extract of the cultivar Valenciana after seven cycles of extraction (70 min); and punicalins, punicalagins, ellagic acid, and total polyphenols showed increases of 47.9%, 45.7%, 45.2%, and 46.1%, respectively, in the by-product extract of the cultivar Wonderful after seven cycles of extraction (70 min).

Comparing the maximum values of phenolic compounds (ellagitannins) and total polyphenols obtained by MAEs to those obtained by conventional extractions, as observed for UAEs, the MAEs increased the extraction yields with respect to lab-scale extractions. Specifically, the increases were 151.6%, 149.7%, 151.9%, and 147.9% for punicalins, punicalagins, ellagic acid, and total polyphenols, respectively, in the by-product extract of the cultivar Acco after five cycles of extraction (50 min); 94.2%, 94.4%, 97.4%, and 93.5% for punicalins, punicalagins, ellagic acid, and total polyphenols, respectively, in the by-product extract of the cultivar Hicaz after five cycles of extraction (50 min); 76.4%, 77.9%, 80.1%, and 77.9% for punicalins, punicalagins, ellagic acid, and total polyphenols, respectively, in the by-product extract of the cultivar Jolly Red after six cycles of extraction (60 min); 195.9%, 183.4%, 192.4%, and 187.0% for punicalins, punicalagins, ellagic acid, and total polyphenols, respectively, in the by-product extract of the cultivar Parfianka after five cycles of extraction (50 min); 137.7%, 135.7%, 132.1%, and 135.5% for punicalins, punicalagins, ellagic acid, and total polyphenols, respectively, in the by-product extract of the cultivar Valenciana after six cycles of extraction (60 min); and 57.0%, 54.6%, 54.0%, and 55.0% for punicalins, punicalagins, ellagic acid, and total polyphenols, respectively, in the by-product extract of the cultivar Wonderful after six cycles of extraction (60 min).

Regarding the extraction of phenolics, when comparing UAEs to MAEs, the results of this study confirm those reported in the literature [6,22,23,24]. In fact, the MAE of phenolics from pomegranate by-products was found to be more efficient and faster than UAE. The maximum power setting of 1.25 KW substantially enhanced the extraction process, resulting in the highest yield of bioactive compounds. This finding underscores the importance of MW power in optimizing the MAE process [25]. In addition, we also considered the comparison with the ultrasound extraction system, which operates at a fixed maximum power of 1.25 KW. Therefore, we aimed to match this power level for a more direct comparison between the two extraction methods. Comparing the maximum values of phenolic compounds (ellagitannins) and total polyphenols obtained by MAEs to those obtained by UAEs, the MAEs exhibited increases in extraction yields ranging from 3.5 to 5.8% for by-product extracts of the cultivars Acco and Valenciana, respectively. Additionally, to achieve the maximum values of phenolic compounds (ellagitannins) and total polyphenols, the extraction cycles were reduced from 6–7 cycles (60–70 min) to 5–6 cycles (50–60 min) for UAEs and MAEs, respectively, depending on the cultivars (the results are presented in Figure 2 and Figure 3 and in the Supplementary Materials (Figures S1 and S2)).

In summary, our results demonstrate that both UAE and MAE are effective techniques for extracting phenolic compounds from pomegranate by-products, with MAE showing slightly higher efficiency under the conditions tested.

### 3.3. Hydrodynamic Cavitation-Based Extractions

Considering the results obtained from lab-scale extractions as well as UAEs and MAEs, the by-product extracts of the Wonderful cultivar consistently exhibited the highest levels of ellagitannins and the highest total polyphenol content among all types of extraction procedures used (conventional or advanced). For these reasons, and given that Wonderful pomegranate is the most widely cultivated variety in the world and is renowned for its high-quality of fruit and juice, being the most processed, and generating high quantities of by-products [26,27], it was selected for the HC extractions.

Hydrodynamic cavitation (HC) is a promising technique for the extraction of bioactive compounds due to the intense local conditions (high temperature and pressure) created by the collapse of cavitation bubbles. These conditions can enhance cell disruption and mass transfer, thereby increasing the yield of extracted compounds [28]. In our study, HC demonstrated superior efficiency in extracting phenolic compounds (ellagitannins) and total polyphenols from pomegranate by-products compared to UAE and MAE. The efficiency of HC observed in our study aligns with findings from other researchers who have explored this technique. HC significantly enhances the extraction of natural products due to the effective mixing and localized hotspots created during cavitation events [29]. Similarly, HC has been shown to extract higher yields of phenolic compounds from plant materials and process food waste more effectively than conventional methods. Specifically, HC significantly improved polyphenol extraction from waste orange peel compared to conventional methods [30]. Our results are consistent with this finding, indicating that HC is effective across different plant matrices for extracting various bioactive compounds. When comparing HC to UAE, our results are consistent with studies that highlight the advantages of UAE in terms of rapid extraction and high yields due to acoustic cavitation [19], probably due to the similar cavitation mechanisms involved in both methods. In comparison to MAE, our results show that HC can achieve similar or slightly higher yields of bioactive compounds. This finding is supported by previous research, which noted that while MAE is highly effective due to rapid heating and efficient energy transfer, HC can provide comparable or better results through intense cavitation effects [20].

**Figure 4:**
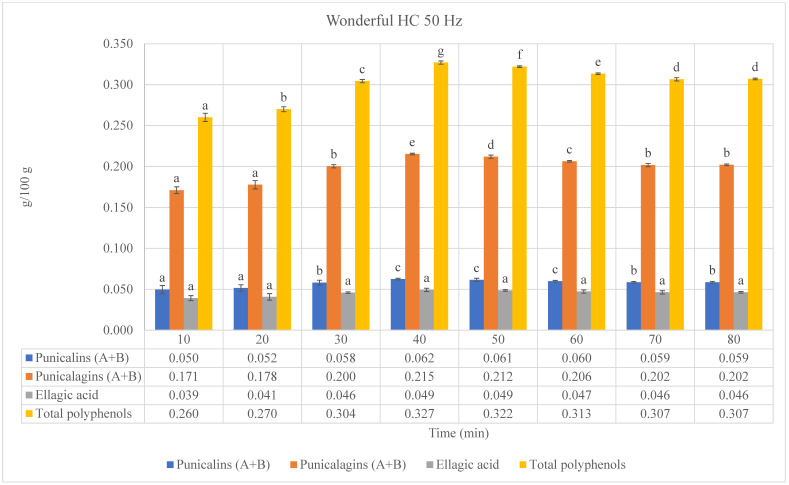

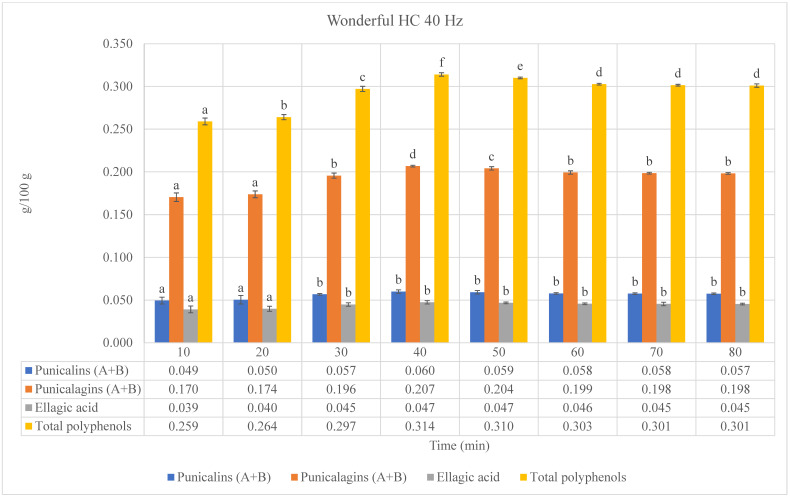

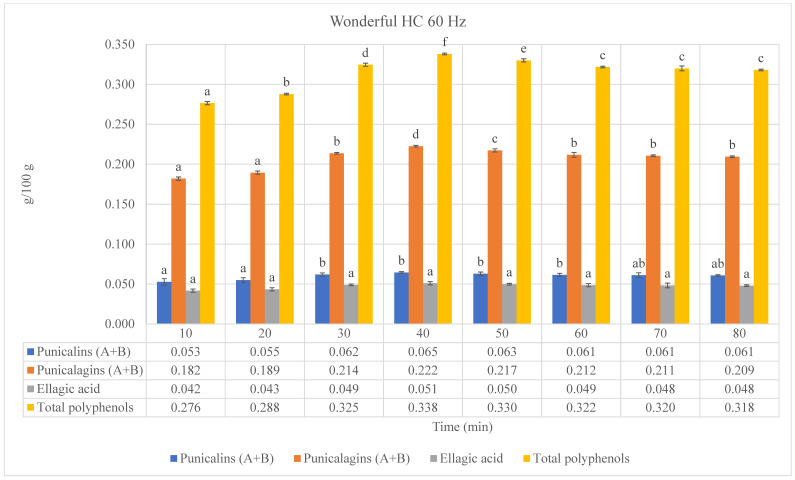
Phenolic compounds (punicalins, punicalagins, and ellagic acid), “a”, and total polyphenols, “b”, of Wonderful pomegranate by-product extracts obtained by hydrodynamic cavitation (HC) performed at different frequencies (40, 50, and 60 Hz). “a”: values expressed as grams of standard compounds per 100 g of extract (g/100 g extract). “b”: values expressed as grams of gallic acid equivalents (GAEs) per 100 g of extract (g GAE/100 g extract). Values are expressed as the mean ± standard deviation (SD). Mean values with different letters (a–g) on the bars denote statistical differences (*p* < 0.05) among different extraction times for both phenolic compounds and total polyphenols.

Three different frequencies (40, 50, and 60 Hz) were utilized during the HC extractions of Wonderful pomegranate by-products (Figure 4). The results reveal that the frequency of hydrodynamic cavitation plays a crucial role in maximizing the extraction efficiency of bioactive compounds [28]. The increase in frequency from 40 to 60 Hz resulted in higher extraction yields, confirming the importance of optimizing cavitation parameters. This finding underscores the importance of tuning hydrodynamic cavitation parameters to the specific physical and chemical properties of the target compounds and source material. According to the literature [30], the results collectively suggest that higher frequencies of hydrodynamic cavitation can lead to a more efficient extraction of bioactive compounds. The data show a clear trend: as the frequency of HC increases from 40 to 60 Hz, the concentration of punicalins, punicalagins, ellagic acid, and total polyphenols also increases. In fact, the highest levels of ellagitannins and the highest total polyphenol content were observed with HC performed at 60 Hz. Specifically, at 60 Hz, HC achieved the highest extraction yields: 0.065, 0.222, and 0.051 g/100 g extract for punicalins, punicalagins, and ellagic acid, respectively, and 0.338 g GAEs/100 g extract for total polyphenols (Table 2). When comparing the maximum values of phenolic compounds (ellagitannins) and total polyphenols obtained through HC at 60 Hz to those obtained through conventional extractions, HC exhibited the highest extraction yields, surpassing both UAEs and MAEs. Specifically, there were increases of 79.2%, 76.6%, 75.9%, and 77.0% for punicalins, punicalagins, ellagic acid, and total polyphenols, respectively, in the by-product extract of the cultivar Wonderful after four cycles of extraction (40 min) performed at 60 Hz. Compared to conventional lab-scale extraction, HC reduced the extraction time by 50% (40 and 80 min for HC and lab-scale extraction, respectively) while significantly increasing the yield, demonstrating its time efficiency and effectiveness. When directly compared with UAEs and MAEs, HC showed increases in extraction yields for by-product extracts of the cultivar Wonderful ranging from 6.1 to 21.1% for frequencies ranging from 40 to 60 Hz, respectively. Moreover, to achieve the maximum values of phenolic compounds (ellagitannins) and total polyphenols, the extraction cycles were reduced from 6–7 cycles (60–70 min) for MAEs and UAEs, respectively, to 4 cycles (40 min) for HC extractions (Table 2). While UAE and MAE are both effective, their performance under the maximum power settings used in this study did not match that of HC. UAE, though beneficial for its lower energy consumption and ability to operate at lower temperatures, was less efficient, possibly due to its dependence on acoustic cavitation, which may not have been as uniformly effective across the plant material. MAE, on the other hand, showed significant extraction efficiency but was slightly less effective than HC, likely due to the limitations of microwave energy penetration and distribution within the plant matrix [18,19]. The efficiency of HC relies on uniform experimental conditions for all tests. These conditions included utilizing the maximum power setting (1.25 KW) for both UAE and MAE, while ensuring that the same experimental conditions (by-products/solvent ratio, extraction time, solvent used, and temperature) were applied in all experiments. This approach allowed us to directly compare the performance of the three extraction techniques under identical operating conditions. The superior performance of HC under these conditions aligns with literature findings that emphasize the efficiency of hydrodynamic cavitation in breaking down cell walls and facilitating the release of bioactive compounds [29]. Our results demonstrate that HC is a highly efficient extraction method for pomegranate by-products, outperforming both UAEs and MAEs. The higher yields of bioactive compounds achieved with HC highlight its potential as a preferred method for industrial-scale extraction processes.

## 4. Conclusions

This research underscores the importance of exploring innovative techniques to maximize resource utilization and minimize waste in the food processing industry. The results of this study indicate that the extraction of bioactive compounds from pomegranate processing by-products can be significantly enhanced through the utilization of advanced extraction technologies. The comparison between conventional lab-scale extractions and ultrasound- and microwave-assisted extractions (UAEs and MAEs, respectively) demonstrated notable improvements in extraction yields, particularly for punicalins, punicalagins, ellagic acid, and total polyphenols. These advancements were observed across various pomegranate cultivars, highlighting the versatility and effectiveness of these methods.

Notably, hydrodynamic cavitation-based extractions (HC) emerged as particularly promising, consistently yielding the highest levels of ellagitannins and total polyphenols, especially when operated at higher frequencies. Compared to conventional extractions, HC exhibited substantial increases in extraction yields for Wonderful pomegranate by-products, surpassing the efficiency of both UAEs and MAEs. Furthermore, HC allowed for shorter extraction times, indicating its potential to streamline extraction processes and reduce energy consumption. Additionally, being a continuous process, HC is more suitable for industrial scale-up compared to UAEs and MAEs.

Overall, these findings underscore the potential of advanced extraction technologies in maximizing the recovery of valuable bioactive compounds from pomegranate processing by-products. By implementing these innovative methods, significant improvements in extraction efficiency can be achieved, paving the way for the development of sustainable and environmentally friendly extraction processes in the food industry. Further research in this area is warranted to optimize extraction parameters and explore the full potential of these technologies in industrial-scale applications.

## Figures and Tables

**Table 1:** Phenolic compounds (punicalins, punicalagins, and ellagic acid), “a”, and total polyphenols, “b”, of different cultivars of pomegranate by-product extracts obtained by lab-scale extractions.

Cultivars	Punicalins (A + B)	Punicalagins (A + B)	Ellagic Acid	Total Polyphenols
Acco	0.019 ± 0.011 ab	0.066 ± 0.011 b	0.015 ± 0.005 ab	0.101 ± 0.012 b
Hicaz	0.027 ± 0.002 c	0.093 ± 0.015 de	0.021 ± 0.003 bc	0.142 ± 0.010 c
Jolly Red	0.031 ± 0.005 cd	0.106 ± 0.017 de	0.024 ± 0.004 cd	0.161 ± 0.012 c
Parfianka	0.010 ± 0.002 a	0.036 ± 0.012 a	0.008 ± 0.005 a	0.054 ± 0.020 a
Valenciana	0.021 ± 0.010 ab	0.073 ± 0.011 c	0.017 ± 0.003 b	0.111 ± 0.013 b
Wonderful	0.036 ± 0.001 d	0.126 ± 0.014 f	0.029 ± 0.002 de	0.191 ± 0.011 d

“a”: values expressed as grams of standard compounds per 100 g of extract (g/100 g extract). “b”: values expressed as grams of gallic acid equivalents (GAEs) per 100 g of extract (g GAEs/100 g extract). Values are expressed as the mean ± standard deviation (SD). Mean values with different letters (a–f) within the same column are statistically different (*p* < 0.05).

**Table 2:** Phenolic compounds (punicalins, punicalagins, and ellagic acid), “a”, and total polyphenols, “b”, of Wonderful pomegranate by-product extracts obtained by different extraction technologies.

Type of Extraction (Time)	Punicalins (A + B)	Punicalagins (A + B)	Ellagic Acid	Total Polyphenols
Lab-scale (80 min)	0.036 ± 0.001 a	0.126 ± 0.014 a	0.029 ± 0.002 a	0.191 ± 0.011 a
UAE (70 min)	0.053 ± 0.001 b	0.184 ± 0.002 b	0.042 ± 0.001 b	0.279 ± 0.001 b
MAE (60 min)	0.057 ± 0.002 b	0.195 ± 0.001 c	0.045 ± 0.002 c	0.296 ± 0.001 c
HC 40 Hz (40 min)	0.060 ± 0.002 c	0.207 ± 0.001 d	0.047 ± 0.002 cd	0.314 ± 0.002 d
HC 50 Hz (40 min)	0.062 ± 0.001 c	0.215 ± 0.001 e	0.049 ± 0.003 d	0.327 ± 0.003 e
HC 60 Hz (40 min)	0.065 ± 0.001 d	0.222 ± 0.001 f	0.051 ± 0.002 e	0.338 ± 0.001 f

“a”: values expressed as grams of standard compounds per 100 g of extract (g/100 g extract). “b”: values expressed as grams of gallic acid equivalents (GAEs) per 100 g of extract (g GAE/100 g extract). Values are expressed as the mean ± standard deviation (SD). Mean values with different letters (a–f) within the same column are statistically different (*p* < 0.05).

## Data Availability

The original contributions presented in the study are included in the article/Supplementary Materials, further inquiries can be directed to the corresponding author.

## Supplementary Materials

The following supporting information can be downloaded at: https://www.mdpi.com/article/10.3390/foods13121793/s1, Figure S1: Phenolic compounds, “a”, and total polyphenols, “b”, of Acco, Hicaz, Jolly Red, Parfianka, and Valenciana pomegranate by-product extracts obtained by ultrasound-assisted extractions (UAEs); Figure S2: Phenolic compounds, “a”, and total polyphenols, “b”, of Acco, Hicaz, Jolly Red, Parfianka, and Valenciana pomegranate by-product extracts obtained by microwave-assisted extractions (MAEs).
