# Supplementary material for: Sustainable Extraction Protocols for the Recovery of Bioactive Compounds from By-Products of Pomegranate Fruit Processing
Source: Foods. 2024 Jun 7;13(12):1793. doi: 10.3390/foods13121793 (PMC11203178; doi:10.3390/foods13121793)
Supplement: Supplementary file 1 [file foods-13-01793-s001.zip › foods-3034640-supplementary.pdf]

foods

SUPPLEMENTARY MATERIAL

Sustainable Extraction Protocols for the Recovery of Bioactive Compounds from By-Products of Pomegranate Fruit Processing

Gabriele Ballistreri *, Margherita Amenta, Simona Fabroni, Nicolina Timpanaro and Giusy Maria Platania

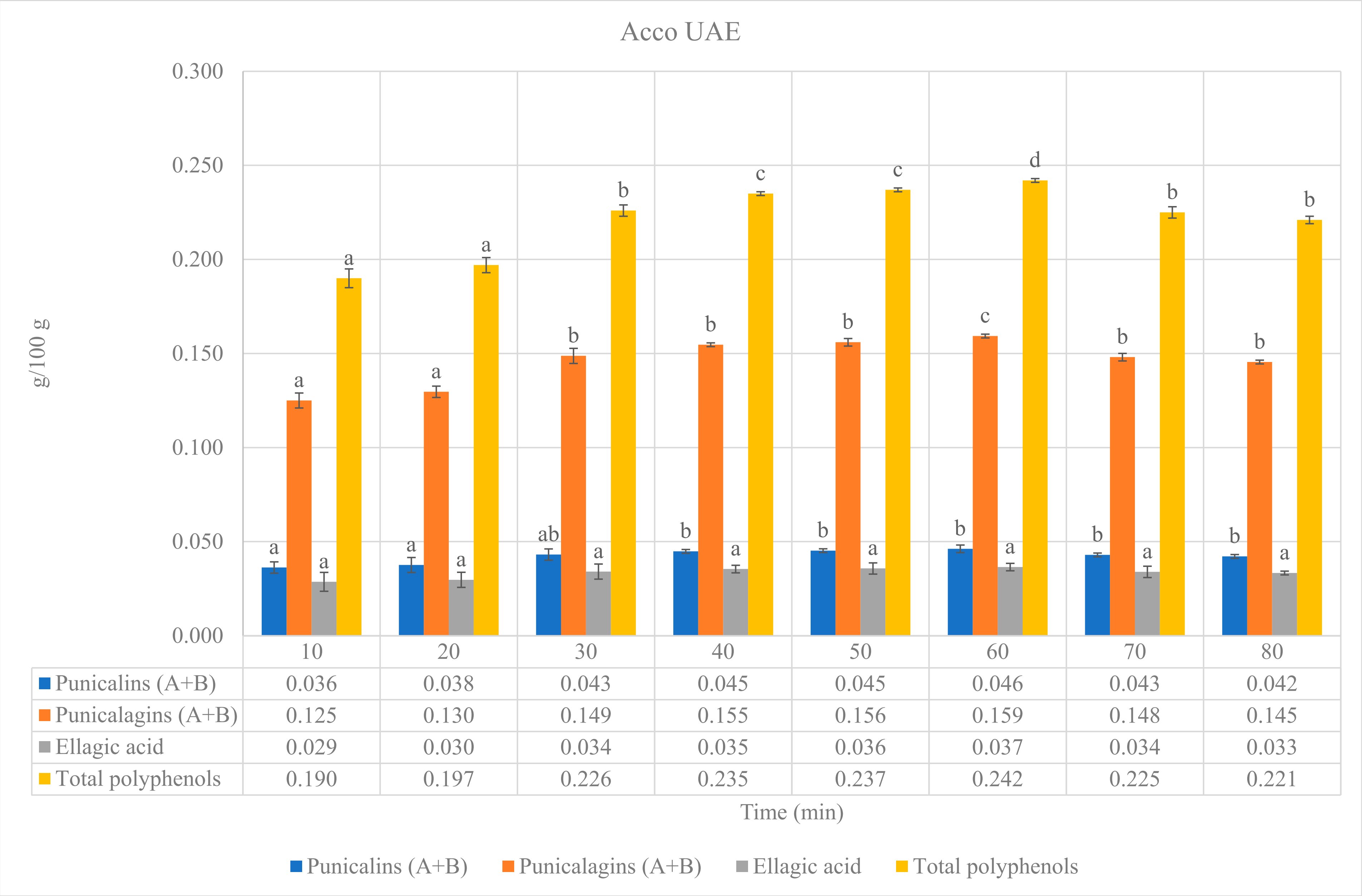

	10	20	30	40	50	60	70	80
Punicalins (A+B)	0.036	0.038	0.043	0.045	0.045	0.046	0.043	0.042
Punicalagins (A+B)	0.125	0.130	0.149	0.155	0.156	0.159	0.148	0.145
Ellagic acid	0.029	0.030	0.034	0.035	0.036	0.037	0.034	0.033
Total polyphenols	0.190	0.197	0.226	0.235	0.237	0.242	0.225	0.221

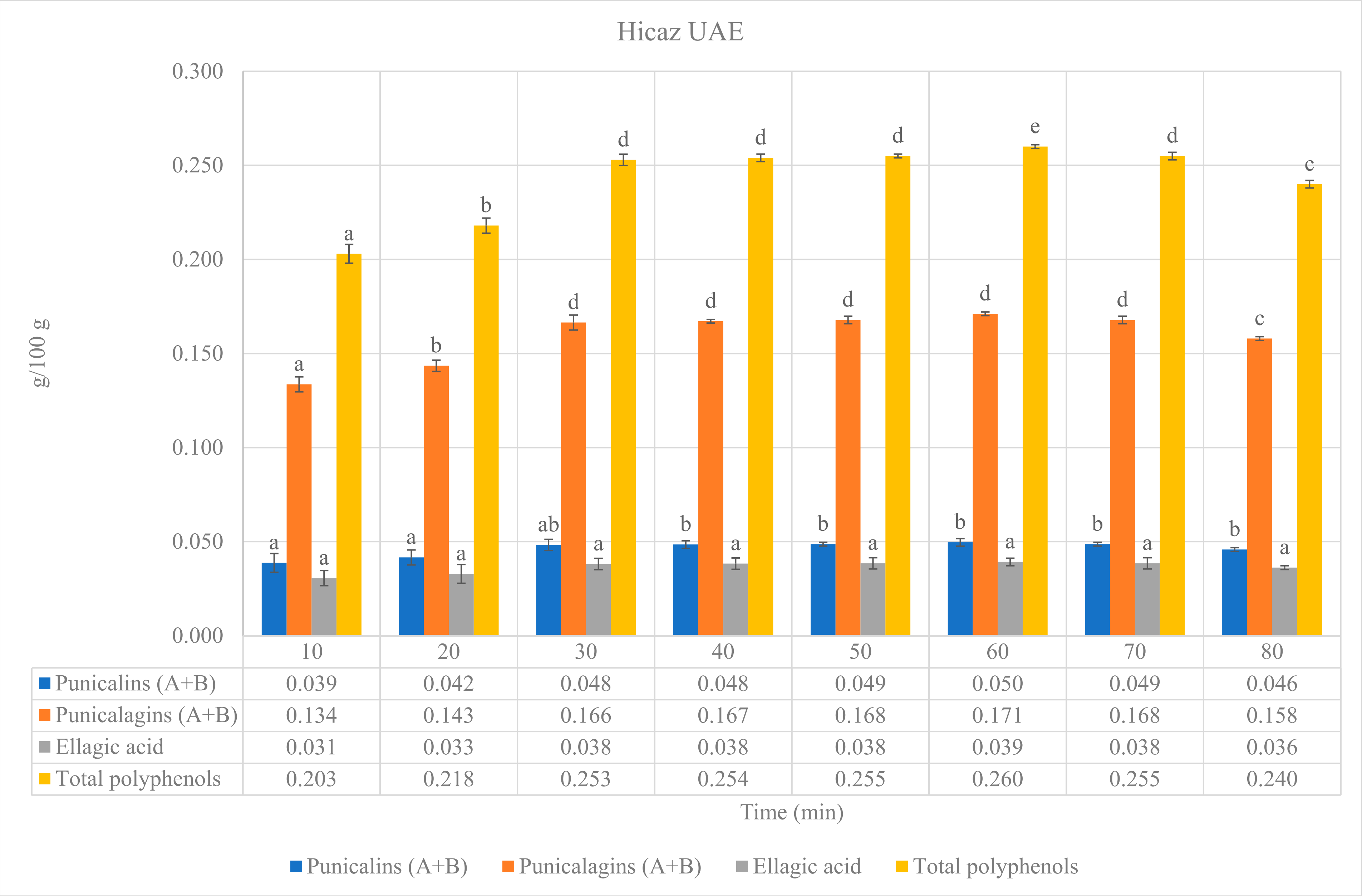

	10	20	30	40	50	60	70	80
Punicalins (A+B)	0.039	0.042	0.048	0.048	0.049	0.050	0.049	0.046
Punicalagins (A+B)	0.134	0.143	0.166	0.167	0.168	0.171	0.168	0.158
Ellagic acid	0.031	0.033	0.038	0.038	0.038	0.039	0.038	0.036
Total polyphenols	0.203	0.218	0.253	0.254	0.255	0.260	0.255	0.240

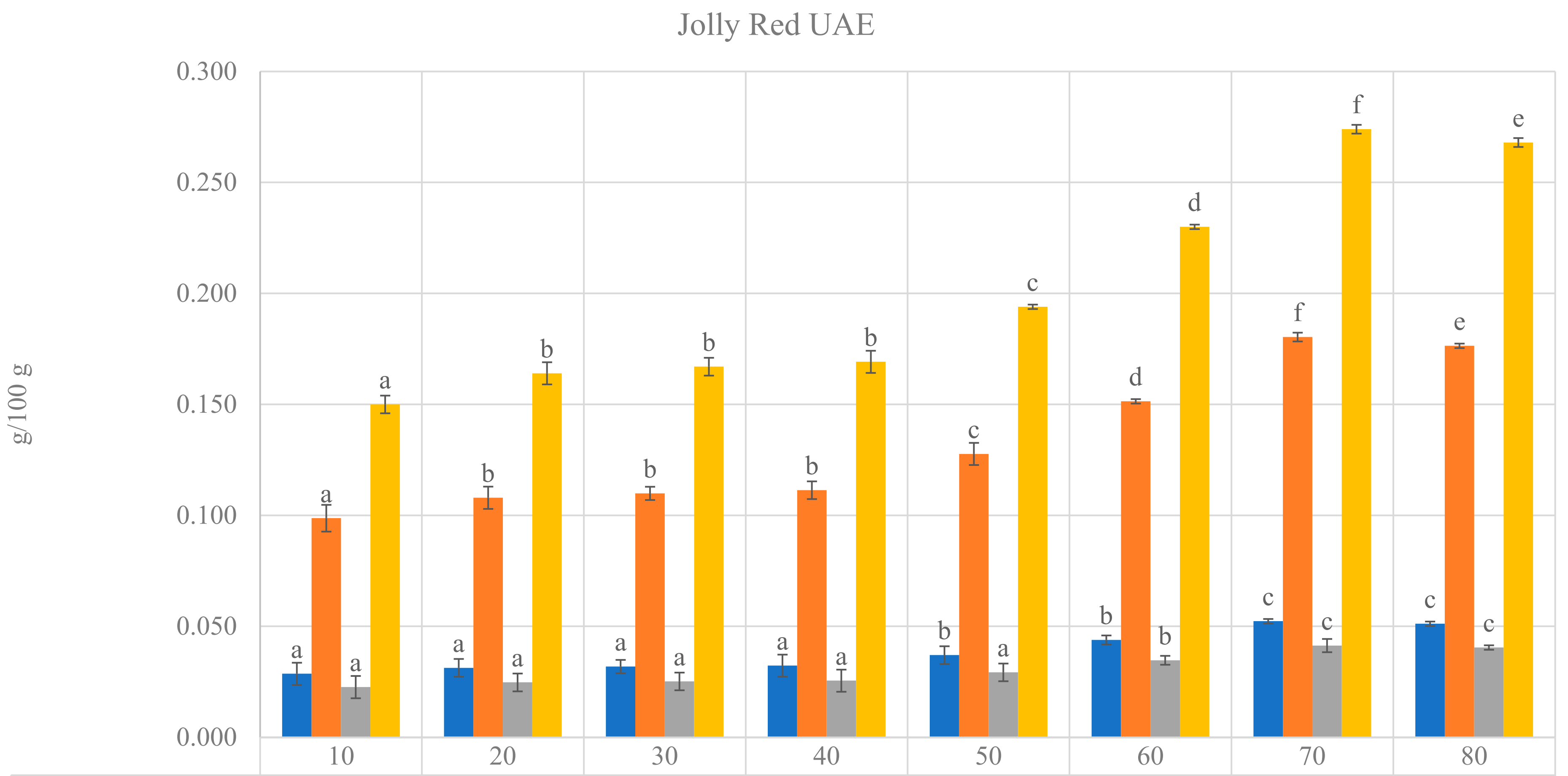

	10	20	30	40	50	60	70	80
Punicalins (A+B)	0.029	0.031	0.032	0.032	0.037	0.044	0.052	0.051
Punicalagins (A+B)	0.099	0.108	0.110	0.111	0.128	0.151	0.180	0.176
Ellagic acid	0.023	0.025	0.025	0.026	0.029	0.035	0.041	0.040
Total polyphenols	0.150	0.164	0.167	0.169	0.194	0.230	0.274	0.268

Time (min)

Punicalins (A+B) Punicalagins (A+B) Ellagic acid Total polyphenols

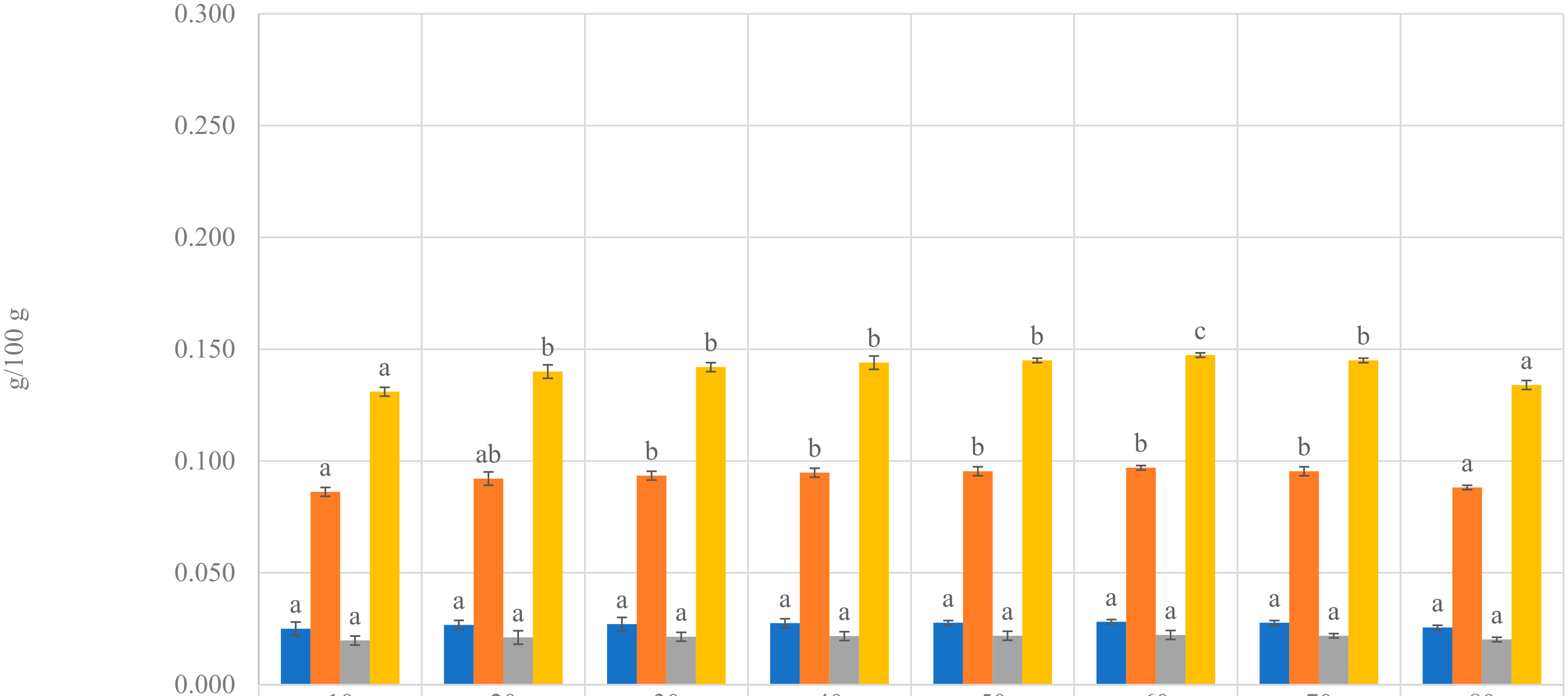

	10	20	30	40	50	60	70	80
Punicalins (A+B)	0.025	0.027	0.027	0.027	0.028	0.028	0.028	0.026
Punicalagins (A+B)	0.086	0.092	0.093	0.095	0.095	0.097	0.095	0.088
Ellagic acid	0.020	0.021	0.021	0.022	0.022	0.022	0.022	0.020
Total polyphenols	0.131	0.140	0.142	0.144	0.145	0.147	0.145	0.134

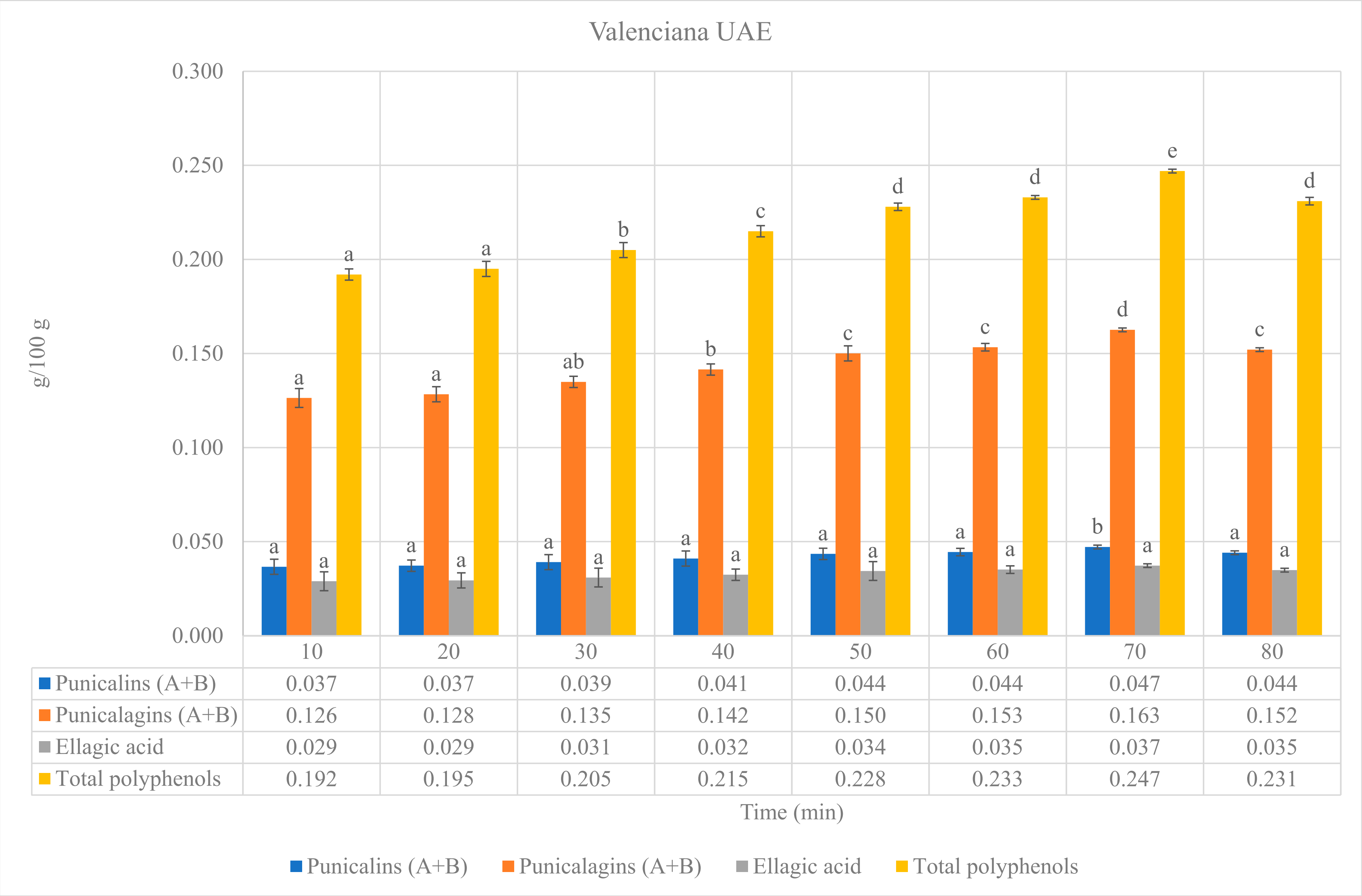

	10	20	30	40	50	60	70	80
Punicalins (A+B)	0.037	0.037	0.039	0.041	0.044	0.044	0.047	0.044
Punicalagins (A+B)	0.126	0.128	0.135	0.142	0.150	0.153	0.163	0.152
Ellagic acid	0.029	0.029	0.031	0.032	0.034	0.035	0.037	0.035
Total polyphenols	0.192	0.195	0.205	0.215	0.228	0.233	0.247	0.231

Figure S1. Phenolic compounds (punicalins, punicalagins and ellagic acid)-"a" and total polyphenols-"b" of Acco, Hicaz, Jolly Red, Parfianka and Valenciana pomegranate by-products extracts obtained by ultrasound-assisted extractions (UAEs).-"a": Values expressed as grams of standard compounds per 100 grams of extract (g/100 g extract). "b": Values expressed as grams of gallic acid equivalents (GAE) per 100 grams of extract (g GAE/100 g extract).

Values expressed as mean ± standard deviation (SD). Mean values with different letters (a–f) on the bars denote statistical differences ($P < 0.05$) among different extraction times for both phenolic compounds and total polyphenols.

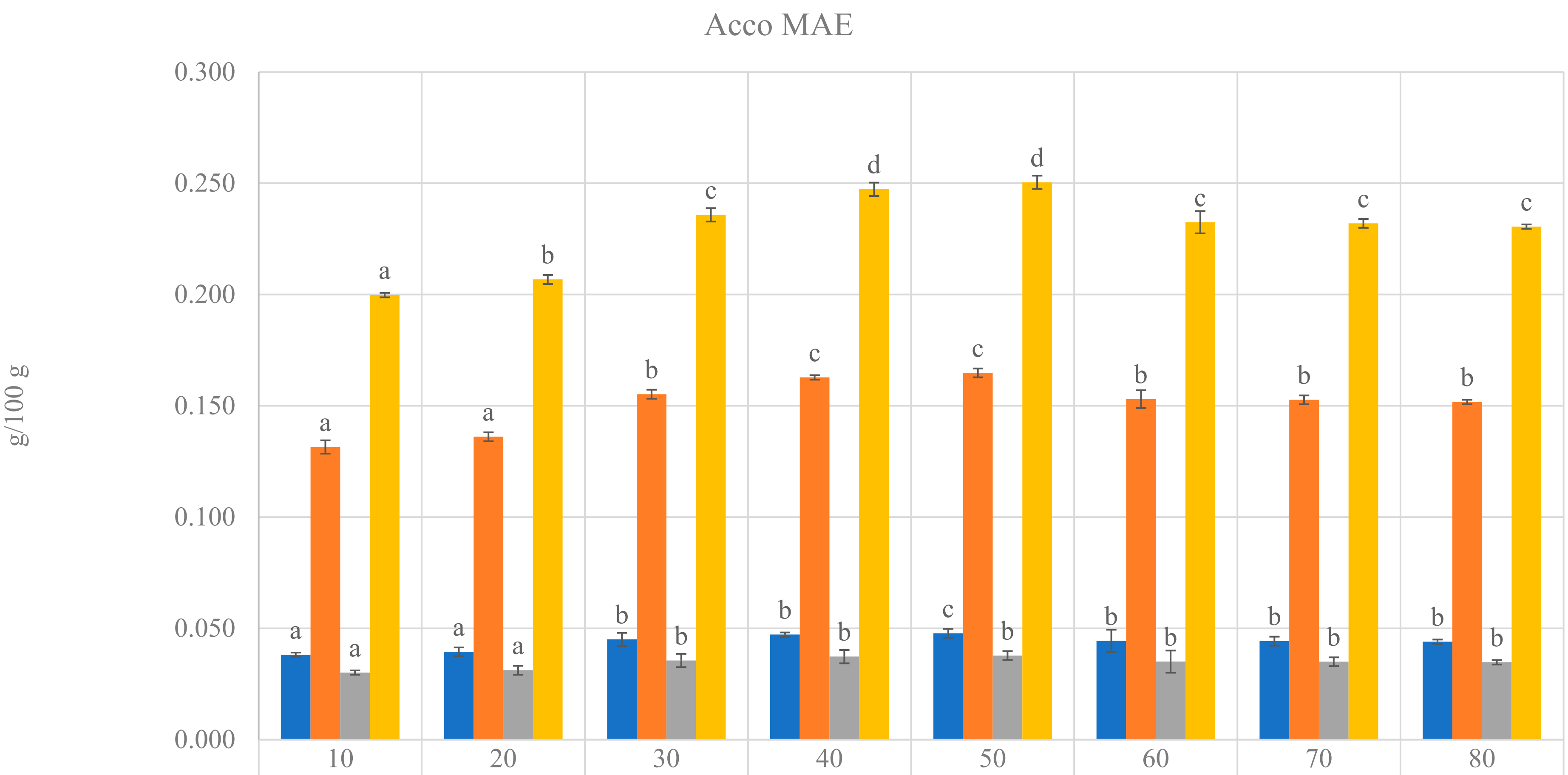

	10	20	30	40	50	60	70	80
Punicalins (A+B)	0.038	0.039	0.045	0.047	0.048	0.044	0.044	0.044
Punicalagins (A+B)	0.131	0.136	0.155	0.163	0.165	0.153	0.153	0.152
Ellagic acid	0.030	0.031	0.036	0.037	0.038	0.035	0.035	0.035
Total polyphenols	0.200	0.207	0.236	0.247	0.250	0.233	0.232	0.231

Time (min)

Punicalins (A+B) Punicalagins (A+B) Ellagic acid Total polyphenols

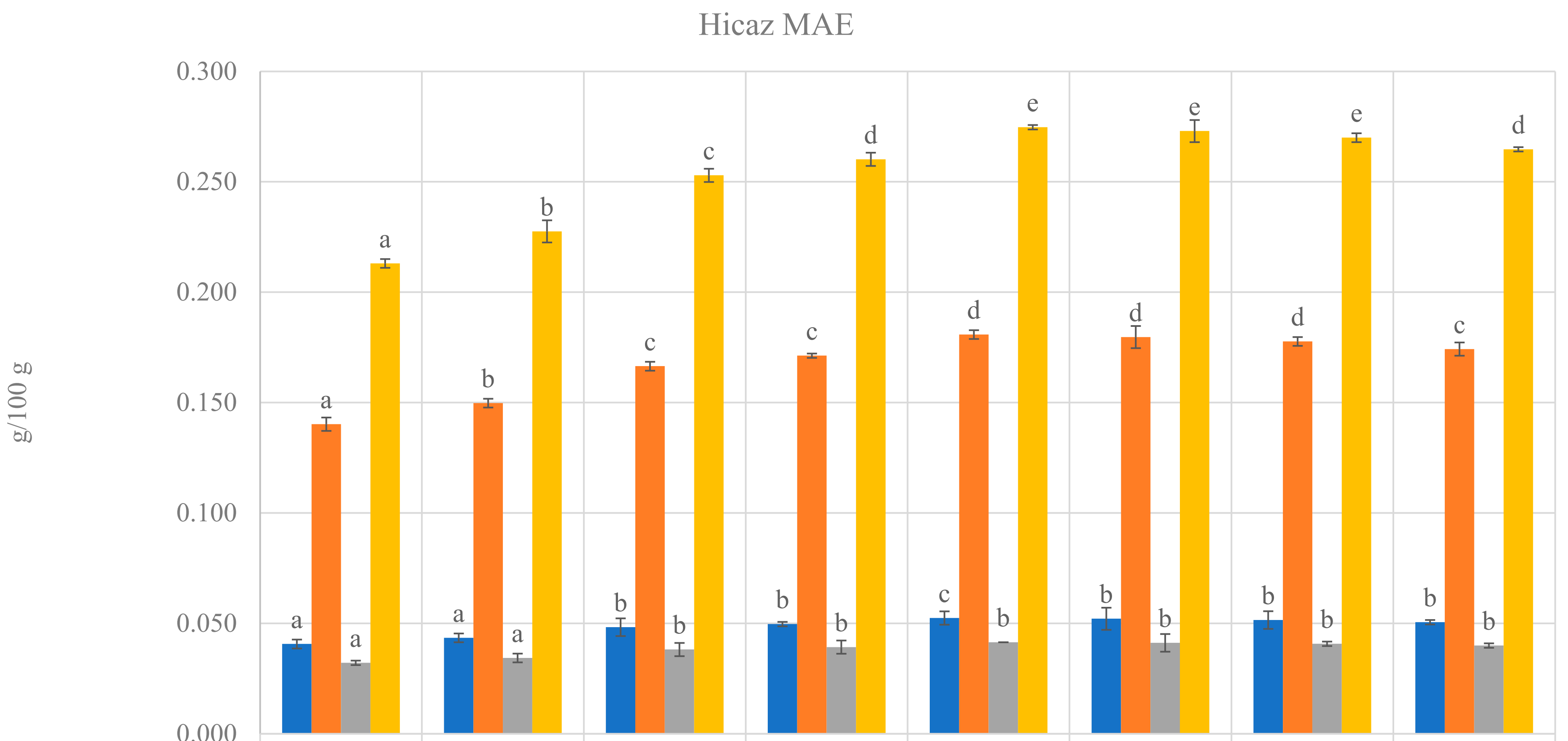

	10	20	30	40	50	60	70	80
Punicalins (A+B)	0.041	0.043	0.048	0.050	0.052	0.052	0.052	0.051
Punicalagins (A+B)	0.140	0.150	0.166	0.171	0.181	0.180	0.178	0.174
Ellagic acid	0.032	0.034	0.038	0.039	0.041	0.041	0.041	0.040
Total polyphenols	0.213	0.228	0.253	0.260	0.275	0.273	0.270	0.265

Time (min)

Punicalins (A+B) Punicalagins (A+B) Ellagic acid Total polyphenols

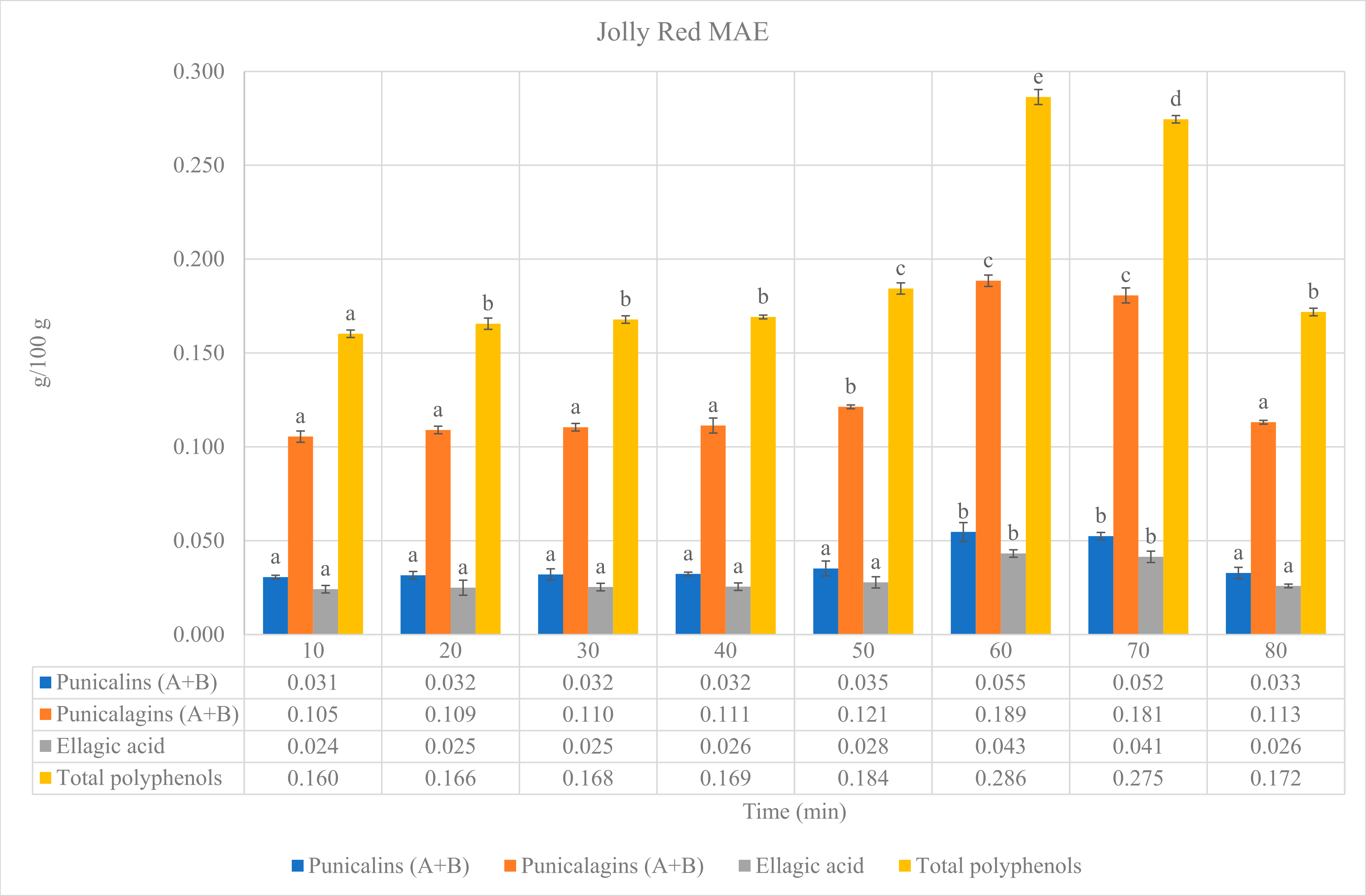

	10	20	30	40	50	60	70	80
Punicalins (A+B)	0.031	0.032	0.032	0.032	0.035	0.055	0.052	0.033
Punicalagins (A+B)	0.105	0.109	0.110	0.111	0.121	0.189	0.181	0.113
Ellagic acid	0.024	0.025	0.025	0.026	0.028	0.043	0.041	0.026
Total polyphenols	0.160	0.166	0.168	0.169	0.184	0.286	0.275	0.172

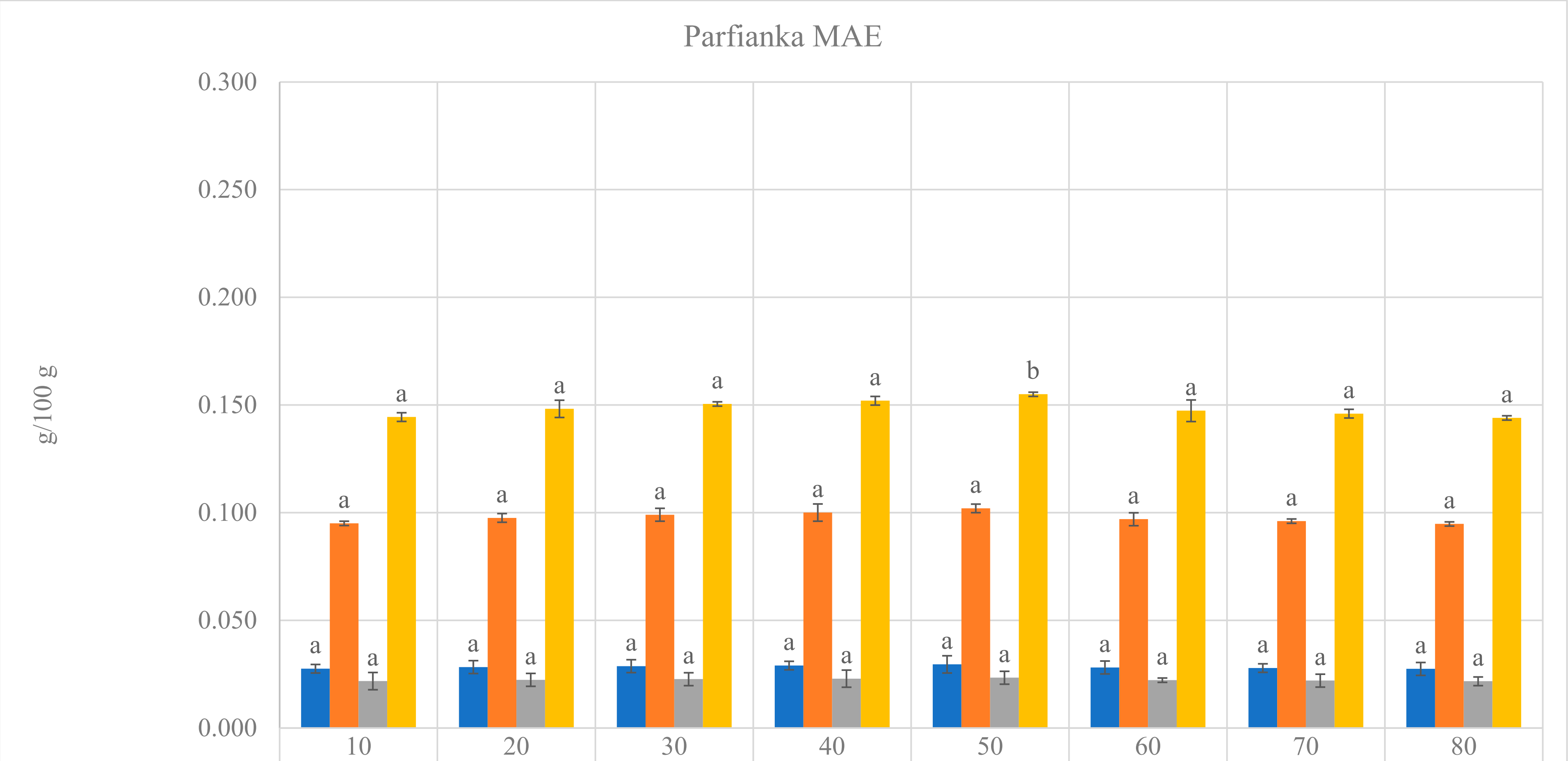

	10	20	30	40	50	60	70	80
Punicalins (A+B)	0.028	0.028	0.029	0.029	0.030	0.028	0.028	0.027
Punicalagins (A+B)	0.095	0.098	0.099	0.100	0.102	0.097	0.096	0.095
Ellagic acid	0.022	0.022	0.023	0.023	0.023	0.022	0.022	0.022
Total polyphenols	0.144	0.148	0.150	0.152	0.155	0.147	0.146	0.144

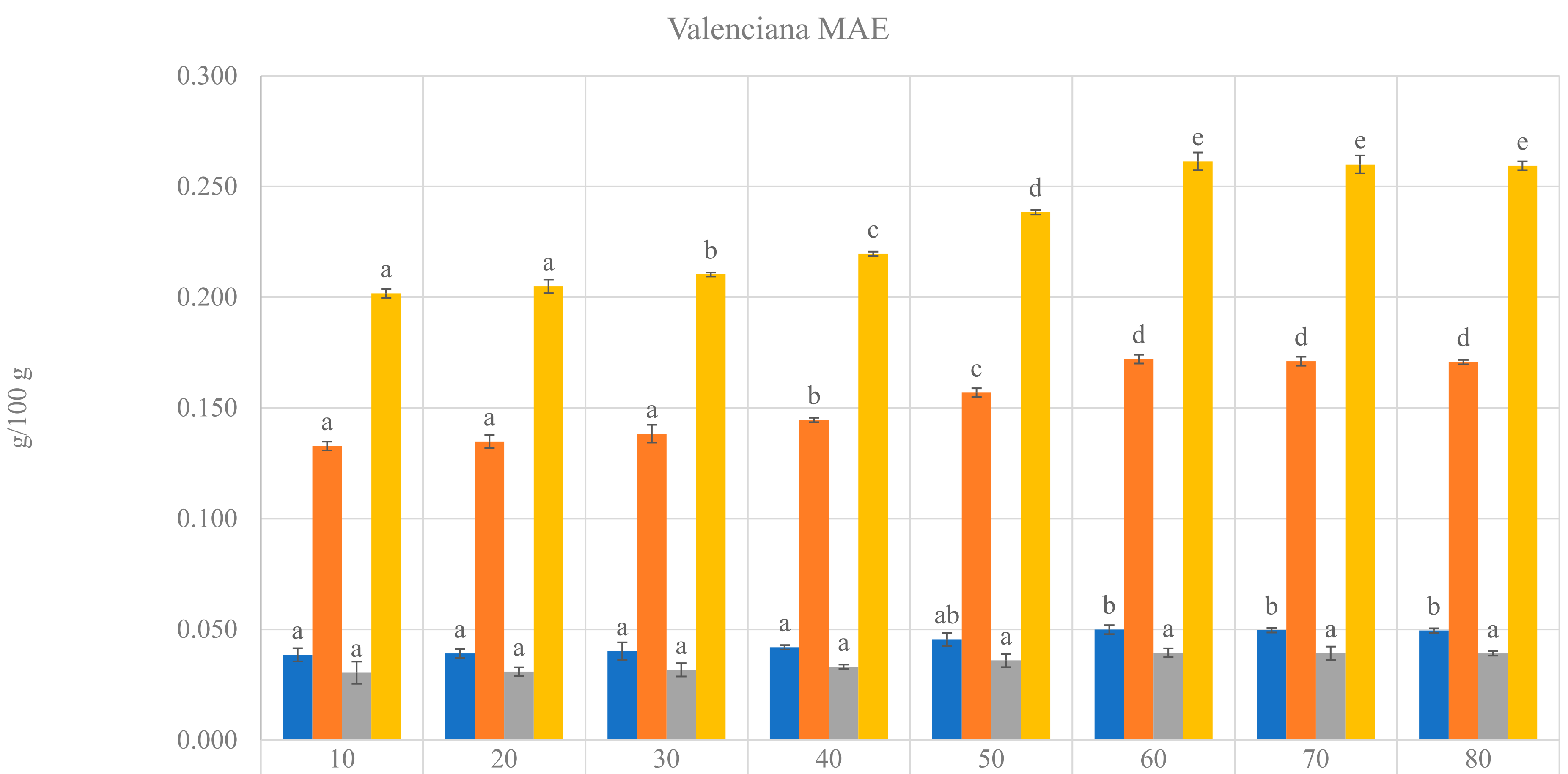

Time (min)	10	20	30	40	50	60	70	80
Punicalins (A+B)	0.039	0.039	0.040	0.042	0.046	0.050	0.050	0.050
Punicalagins (A+B)	0.133	0.135	0.138	0.145	0.157	0.172	0.171	0.171
Ellagic acid	0.030	0.031	0.032	0.033	0.036	0.039	0.039	0.039
Total polyphenols	0.202	0.205	0.210	0.220	0.238	0.261	0.260	0.259

Time (min)

Punicalins (A+B) Punicalagins (A+B) Ellagic acid Total polyphenols

Figure S2. Phenolic compounds (punicalins, punicalagins and ellagic acid) "a" and total polyphenols "b" of Acco, Hicaz, Jolly Red, Parfianka and Valenciana pomegranate by-products extracts obtained by microwave-assisted extractions (MAEs). "a": Values expressed as grams of standard compounds per 100 grams of extract (g/100 g extract). "b": Values expressed as grams of gallic acid equivalents (GAE) per 100 grams of extract (g GAE/100 g extract).

Values expressed as mean ± standard deviation (SD). Mean values with different letters (a–e) on the bars denote statistical differences ($P < 0.05$) among different extraction times for both phenolic compounds and total polyphenols.
